# Keratin Promotes Differentiation of Keratinocytes Seeded on Collagen/Keratin Hydrogels

**DOI:** 10.3390/bioengineering9100559

**Published:** 2022-10-15

**Authors:** Kameel Zuniga, Neda Ghousifam, John Sansalone, Kris Senecal, Mark Van Dyke, Marissa Nichole Rylander

**Affiliations:** 1 Department of Biomedical Engineering, The University of Texas at Austin, Austin, TX 78712, USA; 2 Department of Mechanical Engineering, The University of Texas at Austin, Austin, TX 78712, USA; 3 Natick Soldier Center, U.S. Army Soldier & Biological Chemical Command, Natick, MA 01760, USA; 4 College of Biomedical Engineering, The University of Arizona, Tucson, AZ 85721, USA

**Keywords:** keratinocytes, differentiation, collagen, keratin, hydrogel, lysosome, glucocerebrosidase, involucrin

## Abstract

Keratinocytes undergo a complex process of differentiation to form the stratified stratum corneum layer of the skin. In most biomimetic skin models, a 3D hydrogel fabricated out of collagen type I is used to mimic human skin. However, native skin also contains keratin, which makes up 90% of the epidermis and is produced by the keratinocytes present. We hypothesized that the addition of keratin (KTN) in our collagen hydrogel may aid in the process of keratinocyte differentiation compared to a pure collagen hydrogel. Keratinocytes were seeded on top of a 100% collagen or 50/50 C/KTN hydrogel cultured in either calcium-free (Ca-free) or calcium+ (Ca+) media. Our study demonstrates that the addition of keratin and calcium in the media increased lysosomal activity by measuring the glucocerebrosidase (GBA) activity and lysosomal distribution length, an indication of greater keratinocyte differentiation. We also found that the presence of KTN in the hydrogel also increased the expression of involucrin, a differentiation marker, compared to a pure collagen hydrogel. We demonstrate that a combination (i.e., containing both collagen and kerateine or “C/KTN”) hydrogel was able to increase keratinocyte differentiation compared to a pure collagen hydrogel, and the addition of calcium further increased the differentiation of keratinocytes. This multi-protein hydrogel shows promise in future models or treatments to increase keratinocyte differentiation into the stratum corneum.

## 1. Introduction

Keratinocytes undergo a complex process of differentiation to develop the cornified layer that protects the body from foreign invasion. Because the epidermis is continually renewing every two weeks, keratinization requires a balance between proliferation, differentiation, and apoptosis [1]. This involves major changes in the intracellular organization of the keratinocytes, and the subsequent development of the stratum corneum. In the basal layer, the keratinocytes are in a proliferative state and express cell-specific markers, such as cytokeratin 14 (K14) [1,2,3,4]. These epidermal stem cells give rise to daughter cells that migrate up into the spinous layer [3], and as they detach from the basal layer, they become stratified as the calcium concentration increases. Seo et al. discovered that 290 genes are upregulated, most of which are related to differentiation, in response to increased calcium [5]. These genes include involucrin and the keratin 1 gene, which includes the activator 1 (AP1) protein, a calcium-responsive promoter [6,7,8]. In addition, the G-protein calcium-sensing receptor (CaSR) allows keratinocytes to sense the levels of extracellular calcium, allowing the cells to differentiate based on the existing extracellular calcium gradient found in the epidermal layer, and leading to increased intracellular calcium levels [9]. Although the mechanism of why calcium regulates keratinocyte differentiation is not fully understood, the calcium gradient throughout the epidermis allows sequential differentiation through outside–in and inside–out signaling of calcium, allowing the formation of desomosomes, adherens junctions, and tight junctions for stratification [4]. These membrane complexes trigger changes in actin distribution and further increase intracellular calcium, increasing the expression of differentiation markers such as involucrin, a component of the cornified envelope of the epidermis [4,10,11,12].

Literature shows that lysosomal activity is a requirement in keratinocyte differentiation and cornification in 3D in vitro models by triggering mitochondrial metabolism with reactive oxygen species production, which causes autophagy lysosomal degradation, creating a feedback loop [13]. As the nucleus disappears and cells flatten, keratinocytes become dead corneocytes. The corneocytes are mechanically strong due to the crosslinking of the keratin networks; moreover, because they have lost their organelles, it is hypothesized that the flattening and volume decrease exhibited by these cells lead to further mechanical resilience by extracellular lipid organization [3], creating a hardened structure of the stratum corneum.

Two-dimensional cell monolayers do not recapitulate the complex 3D architecture of tissue or the inherent cell–cell and cell–matrix interactions critical for understanding responses to injury and wound healing. Most 3D in vitro models consist of an extracellular matrix seeded with fibroblasts, in which keratinocytes are cultured on top. They also consist of an air–liquid interface (ALI), another stimulant for keratinocyte differentiation, which is attained by exposing seeded keratinocytes to the air without being submerged in culture media. This can be accomplished using an organotypic transwell skin model [14,15,16]. The extracellular matrix of 3D in vitro skin models is often in the form of collagen type I hydrogels, and currently, there are no existing skin models that employ keratin. Although keratin does not exist in the dermal layer, it does comprise almost 90% of the epidermis [17]. The addition of keratin in a skin model may promote the differentiation of keratinocytes by forming a cytoskeleton similar to that of native keratinocytes [18]. In native skin, when the keratinocytes flatten, the keratin filaments start to align into disulfide crosslinks and undergo proteolytic degradation [17,19]. Therefore, the presence of keratin in the initial microenvironment may aid in the process of keratinocyte differentiation.

In the current study, we sought to determine whether the inclusion of keratin extracted from human hair in frequently used collagen hydrogels induces the differentiation of keratinocytes, specifically for the telomerase-immortalized keratinocyte cell line (Ker-CT cells) compared to the influence of a pure collagen environment. Although primary keratinocytes are frequently used for modeling skin, telomerase-immortalized keratinocytes engineered by the Rheinwald group by the transduction of primary keratinocytes with the human telomerase reverse transcriptase gene present normal differentiation in monolayer and organotypic skin models [20,21,22,23,24,25,26]. We also chose to employ hair keratin, which is more commercially available, unlike skin. Although both epithelial and hair keratins have a structural subunit containing keratins of differing molecular weight and composition named type I (acidic) and type II (basic–neutral), which interact to form heterodimers and polymerize to form intermediate filaments (IFs), hard keratin is more often used in biomedical research [27,28]. Soft keratins from epithelia exist in the form of loosely packed and disorganized IFs in an amorphous matrix, whereas hair keratins contain a much higher cysteine residue content with IFs organized in an ordered array in an α-keratin matrix, allowing tougher and more stable structures by intermolecular disulfide formation [27,29]. This natural fiber system is similar to synthetic polymers in that it contains structural macromolecules and crosslinkers, making hair keratins more practical and stable for biomedical research.

Our study aims were achieved by creating acellular collagen and collagen/keratin multiprotein hydrogels and growing keratinocytes as a monolayer on top of the gel in calcium-free and calcium+ media. To assess differentiation, lysosomal activity was investigated through a glucocerebrosidase (GBA) assay for both primary keratinocytes and Ker-CT cells, and LysoTracker staining was employed to visualize lysosomes (Ker-CT only). For our initial study of GBA activity only, we also tested the influence of fetal bovine serum (FBS) within the media in addition to calcium (Ca+/FBS), since skin models include FBS in their culture medium to induce differentiation. We also assessed the differentiation of Ker-CT cells by staining specifically for CK14 (proliferation marker) and involucrin (differentiation marker). We were able to demonstrate that the inclusion of keratin in the hydrogel increased lysosomal activity and involucrin expression. Calcium inclusion in the media also further increased the activities of both key markers, indicating higher levels of differentiation. Although we were unable to observe larger cell sizes with the inclusion of keratin, compared to pure collagen, in the hydrogels, the presence of a 3D matrix significantly increased the size of the Ker-CT cells compared to seeding on a 2D plastic surface, indicating the importance of a 3D environment when differentiating keratinocytes.

## 2. Materials and Methods

### 2.1. Collagen Extraction

Collagen type I was isolated from rat tail tendons donated at the conclusion of other studies at the University of Texas Austin (IACUC Protocol ID: AUP-2022-00040). Rat tail tendons were placed in a 1 N HCl solution (Fisher Scientific, Hampton, NH, USA) at approximately at pH 2.0 and stirred for 16 h at room temperature. The solution was then transferred to 50 mL centrifuge tubes (Fisher Scientific, Hampton, NH, USA) and centrifuged at 30,000× *g* (16,000 rpm) for 60 min at 4 °C to pellet the insoluble components. The supernatant was decanted from each tube and transferred into separate 50 mL centrifuge tubes, stored at −20 °C overnight, and freeze dried for 48 h. Samples were dissolved in an appropriate amount of 0.01% glacial acetic acid (Fisher Scientific, Hampton, NH, USA) for a final concentration of 8 mg/mL of stock solution [30].

### 2.2. Kerateine Extraction

Kerateine (KTN) was kindly donated by Dr. Mark Van Dyke, and was extracted from human hair obtained from a commercial source. Briefly, human hair was washed, chopped into small pieces, and soaked in a solution of 0.5 M thioglycolic acid (Sigma-Aldrich, St. Louis, MO, USA) in deionized (DI) water for 12 h at 36 °C with gentle stirring [31]. Hair was then filtered from the liquid with a 500 µm sieve (W. S. Tyler, Mentor, OH, USA), and the reducing solution retained. Free proteins were further extracted in excess 100 mM Tris base for 1 h, followed by DI water for 1 h with gentle shaking at 37 °C. Extracts were collected with the 500 µm sieve and combined with the reductant solution. This entire process was repeated one additional time, and all extracts were combined, centrifuged, and filtered. The combined extracts were then purified and concentrated using tangential flow filtration, frozen, and lyophilized on a Labconco benchtop freeze drying system under ambient conditions.

### 2.3. Cell Culture

Both keratinocyte cell lines were seeded in T-75 Eppendorf HEPA-filtered flasks (Eppendorf, Hamburg, Germany). Primary normal human epidermal keratinocytes (NHEKs, passage 3–6, PromoCell, Heidelberg, Germany) and telomerase-immortalized epidermal keratinocytes (Ker-CT cells, passage 30–40, ATCC, Manassas, VA, USA) were cultured in complete proliferation media composed of keratinocyte growth medium 2 (PromoCell, Heidelberg, Germany) supplemented with 0.004 mL/mL bovine pituitary extract (BPE), 0.125 ng/mL recombinant human epidermal growth factor (EGF), 5 µg/mL recombinant human insulin, 0.33 µg/mL hydrocortisone, 0.39 µg/mL epinephrine, 10 µg/mL recombinant human transferrin, and 0.06 mM CaCl_2_. This media formulation will hereafter be described as Ca-free media. When assessed for differentiation, cells were cultured in proliferation media formulation, without BPE and EGF, with 2.5 mM CaCl_2_ and 0.05 µg/mL ascorbic acid, and referred to as Ca+ media. For the GBA assay, we also included 10% fetal bovine serum (FBS) to the Ca+ media for testing, referred to as Ca+/FBS media. Cells were maintained in 5% CO_2_ atmosphere at 37 °C in a sterile cell culture incubator, and corresponding complete media was changed every 2 days. Cells were detached from the flask by washing with 1× phosphate buffered saline (PBS) and replaced with 4 mL of accutase (PromoCell, Heidelberg, Germany), followed by incubation for 15 min. Detached cell solution was neutralized with complete media, transferred to a 15 mL conical tube, and centrifuged at 200× *g* for 3–5 min. The pellet was isolated and resuspended in 1 mL of complete medium for cell counting.

### 2.4. Collagen/Kerateine Hydrogel Fabrication and Cell Seeding

To mimic healthy skin tissue, 4 mg/mL collagen solution was used [32,33,34,35]. The 100% collagen hydrogels were prepared from 8 mg/mL collagen stock solution and mixed and neutralized with 10× DMEM (Sigma-Aldrich, St. Louis, MO, USA), 1× DMEM (Gibco™, Gaithersburg, MD, USA), and 1 N NaOH (Fisher Scientific, Hampton, NH, USA) for a resulting pH of 7.4. The 50/50 w%/w% C/KTN hydrogels were prepared by combining stock collagen and KTN dissolved in neutralizing buffer of equal volume. Previously in our laboratory, we tested both 50/50 and 30/70 C/KTN hydrogels, and observed low proliferation and growth of fibroblasts in 30/70 C/KTN hydrogels, while cell growth was observed for 50/50 C/KTN hydrogels, comparable to 100% collagen hydrogels [36]. Therefore, we only tested 50/50 C/KTN against 100% collagen hydrogels. Table 1 describes the different hydrogels that were produced, with corresponding KTN concentration and total protein (collagen and keratin) concentration as w%/v%. Stock collagen and KTN/neutralizing buffer solution were combined at a 1:1 v:v ratio and mixed thoroughly with a spatula and positive displacement pipette.

**Figure 1:**
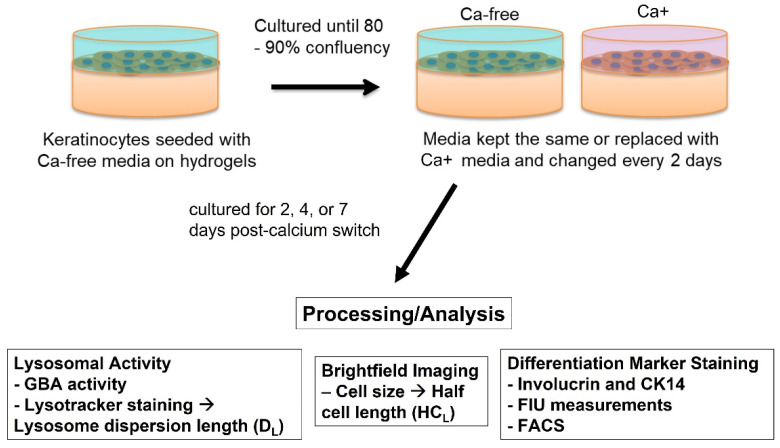
Schematic and flow chart of studies with keratinocytes seeded on either the well plate or on hydrogels. Calcium switch was utilized after reaching 80–90% confluency and then media was changed every 2 days. Half of the samples were cultured in Ca-free media. Keratinocytes were analyzed for lysosomal activity, cell size, and differentiation marker expression.

Samples of 40, 80, or 800 µL of each hydrogel formulation were seeded into 96-, 48-, or 6-well plates, respectively, and allowed to polymerize for 1 h. After polymerization, hydrogels were rinsed three times with 1× PBS for 5 min each. Approximately 0.012–0.02 × 10^6^ cells/cm^2^ was seeded on top of each hydrogel. Cells were also seeded as a blank sample on top of the plate itself at 0.004 × 10^6^ cells/sample. After reaching 80–90% confluency in the plastic well sample, half of the samples were replaced with Ca-free media, and the other half was replaced with Ca+ media, to serve as the “calcium switch” used for differentiating keratinocytes, as shown in Figure 1. For the GBA assay, an extra set of samples were made and replaced with Ca+/FBS media. Tests were conducted at days 2, 4, and 7 post-calcium switch.

### 2.5. Cell Viability Assay

Cell viability of keratinocytes (NHEK and Ker-CT cells) cultured on hydrogels and plastic was determined to normalize subsequent assay results (Figure S1). The CellTiter-Blue^®^ cell viability assay (Promega, Madison, WI, USA) was used to measure cell viability of each sample at each time point. Briefly, each sample media was replaced with 100 µL of fresh proliferation media and 20 µL of CellTiter-Blue^®^ reagent. Cell viability was also performed on cell dilutions of 0.0025 × 10^6^–0.08 × 10^6^ cells for the standard curve. Samples were incubated for 2 h, and the solution for each sample was subsequently transferred to a clean separate well, after which fluorescence intensity was measured with a plate reader at an emission of 560 nm and excitation at 590 nm. Background fluorescence intensity (blank well with media and CellTiter-Blue^®^ reagent) was subtracted from sample readings. Cell number was determined with the standard curve.

### 2.6. Glucocerebrosidase (GBA) Assay

An increase in the differentiation of keratinocytes is directly correlated with an upregulation in lysosome activity [13,37,38]. Following a protocol by Mahanty et al., a GBA assay was conducted on both NHEK and Ker-CT cells seeded on top of the hydrogels or on a blank well plate. GBA is a lysosomal enzyme that assists with the breakdown of glucocerebroside into glucose and ceramide [39]. Complete lack of GBA results in fatal skin abnormalities with reduced barrier formation [40,41,42]. This GBA activity can be observed through the stratum corneum with the accumulation of lysosomal bodies, and therefore is present throughout differentiated keratinocytes [37]. For this initial study, we compared results of Ker-CT cells to primary keratinocytes to determine whether lysosomal activity was increased. Research shows that immortalized cell lines such as HaCaT cells are constantly undergoing lysosomal biogenesis compared to primary cells [43,44]. Therefore, it was critical to compare the results to primary cells to detect differences in GBA activity with increased differentiation. The assay was performed by incubating each sample with 50 µL of 3 mM 4-methylumerlliferyl β-D-glucopyranoside (MUD) dissolved in 0.2 M sodium acetate buffer at pH 4.0 for 3 h at 37 °C. After incubation, the assay was halted by adding 0.2 M glycine buffer at pH 10.8 and incubated for 5 min at room temperature. The solution was removed from each sample into a clean 96-well plate and fluorescence intensity of liberated MUD was read at Ex/Em of 365/445 nm using the Cytation3 Multi-Mode Reader (BioTek Instruments, Winooski, VT, USA). Intensity readings were normalized to the average cell number determined by the cell viability assay.

### 2.7. Lysotracker Staining

To confirm that the KTN was increasing the differentiation of the immortalized keratinocyte cell line, the dispersion length of the lysosomes was measured by lysosomal staining. Keratinocyte differentiation can lead to the dispersion of lysosomes, as increased lysosomal biogenesis occurs in response to the increased intracellular calcium levels. To avoid programmed apoptosis, membrane-bound organelles are likely to accommodate for the excess intracellular calcium, leading to lysosomal dispersion [37,45,46]. To visualize and quantify this dispersion of lysosomes, Ker-CT cells cultured in Ca-free and Ca+ media seeded on top of a plastic plate or a 100% collagen or C/KTN hydrogel were stained with LysoTracker Red DND-99 dye (Thermo Fisher Scientific, Waltham, MA, USA) according to the manufacturer’s protocol. Briefly, the 1 mM probe stock solution was diluted to 75 nM in Ca-free medium. Subsequently, 200 µL of the solution was added to 48-well plates and incubated for 30 min at 37 °C. Samples were subsequently rinsed with Ca-free medium and then fixed with 4% PFA for 10 min at room temperature. Samples were then rinsed three times with 1× PBS and stored at 4 °C until imaging or measurement of fluorescence intensity.

Fluorescence intensity was measured with the Cytation3 Multi-Mode Plate Reader at an Ex/Em of 577/590 nm. Intensity readings were normalized to the average cell number determined by the cell viability assay. Samples were also imaged with the Cytation3 Multi-Mode Imaging System to measure the lysosome dispersion half-cell length of the cells with brightfield (BF) imaging and fluorescence imaging at Ex/Em of 532/588 nm. The dispersion length (D_L_) was quantified by ImageJ software by measuring the distance from the edge of the cell nucleus to the farthest lysosome visualized. The D_L_ of 20 random cells from each sample was measured for day 2 and 4 time points. Similarly, ImageJ software was utilized to determine the half-cell length (HC_L_) by measuring the distance from the edge of the cell nucleus to the cell periphery. The HC_L_ of 20 random cells from each sample was measured for day 2 and 4 time points.

### 2.8. Differentiation Marker Staining

Samples at days 2 and 4 post-calcium switch were stained and the fluorescence intensity was quantified for cytokeratin 14 (CK14), involucrin, and caspase 14 differentiation markers to visualize and quantify the level of differentiation. Samples were fixed with 4% paraformaldehyde for 20 min and subsequently washed three times with 1× PBS. Samples were blocked with 5% FBS for 1 h and then incubated with anti-CK14 (ab181595, Abcam, Cambridge, UK), anti-involucrin (ab68, Abcam, Cambridge, UK), and anti-caspase 14 (ab174847, Abcam, Cambridge, UK) at 1:1000, 1:1000, and 1:250 dilution, respectively, in 5% FBS overnight at 4 °C. The following day, samples were rinsed with 1× PBS three times, and stained with either goat anti-rabbit AF555 (Invitrogen) or anti-mouse AF488 secondary antibody (Invitrogen) at 1:2000 dilution in 5% FBS for 1 h at room temperature.

Stained samples were imaged with the Cytation3 Imaging Plate Reader. Gain and intensity were kept constant between different samples and time points for the RFP (involucrin, CK14, LysoTracker) and the DAPI signal for post-processing analysis. Samples were imaged at 10 and 20× magnification, and fluorescence intensity was measured using ImageJ software. Two methods were used to quantify the intensity of involucrin and CK14 by measuring the fluorescence intensity per cell (corrected total cell fluorescence (CTCF)) and the total fluorescence intensity per image. The corrected total fluorescence intensity per cell was calculated by measuring the integrated density (total fluorescence intensity) of the cell by creating a boundary of one cell with a total of 10 cells per image (total of three images/group, n = 30) and subtracting the integrated intensity by the area of the selected cell multiplied by the mean fluorescence of the background intensity as follows:

(1) CTCF=Integrated Intensity−(Area of Cell ×Background Mean Fluorescence)

This value was then normalized to the CTCF of the DAPI intensity per cell.

With the secondary method, the total fluorescence intensity per image of either the CK14 or involucrin was measured through ImageJ software and normalized to the DAPI cell count/image of 10× magnification images (n = 3).

### 2.9. Flow Cytometry

To acquire sufficient cells for flow cytometry to determine the percentage of keratinocytes expressing differentiation markers, hydrogel samples were prepared in six-well plates and polymerized as previously described. Once polymerized, Ker-CT cells were seeded on top of the hydrogels and allowed to proliferate in Ca-free media until reaching 80–90% confluency. Once reaching confluency, media was replaced with either fresh Ca-free or Ca+ media. After 4 days of culture, cells were isolated by detaching with accutase for Ca-free samples or with 7 mg/mL dispase in 1× HBSS for Ca+ samples. Cells were washed with complete media and then centrifuged to isolate the cell pellet. Cells were passed through a 40 µm cell strainer, strained twice and vortexed.

Once isolated, cells were fixed in 1% paraformaldehyde (PFA) for 10 min on ice. Cells were centrifuged, rinsed in 1× PBS, and treated with 0.05% Triton X for 2 min, and subsequently centrifuged and washed with 1× PBS. Cells were blocked in 5% bovine serum albumin (BSA) for 30 min and subsequently incubated overnight at 4 °C with either anti-involucrin mouse monoclonal antibodies (Abcam, ab68, 1:100 dilution) or anti-cytokeratin 14 rabbit monoclonal antibodies (Abcam, ab181595, 1:190 dilution) in 5% BSA. Samples were also prepared the same way for the isotype controls and incubated with mouse IgG_1_ isotype control (R&D Systems, MAB002) and rabbit IgG isotype controls at the same concentrations as the anti-involucrin and anti-cytokeratin 14 antibodies, respectively. The next day, cells were washed twice in 2% BSA and incubated for 1 h with goat anti-mouse Alexa Fluor 488 secondary antibody (Invitrogen, Waltham, MA, USA) at 1:2000 dilution and goat anti-rabbit phycoerythrin (PE) secondary antibody (Invitrogen, Waltham, MA, USA) at 1:1000 dilution in 5% BSA for 30 min. Cells were washed twice in 2% BSA and stored at 4 °C until analysis.

Analysis was performed with the FACSAria flow cytometer (BD Biosciences, Franklin Lakes, NJ, USA); forward and side scatter were adjusted using unstained control cells for each group to isolate and remove the cell debris from analysis, and the autofluorescence was eliminated from sample cells using the unstained controls by adjusting the signal outputs. At least 50,000 events were collected and analyzed for each marker to determine the percentage of positively stained cells.

### 2.10. Statistical Analysis

The D_L_, HC_L_, and mean fluorescence intensity of differentiation markers/cell are presented as average ± SEM. All other data are presented as average or weighted average ± SD. A two-way ANOVA was performed on GraphPad Prism software. Post hoc analysis was completed with a Tukey’s multiple comparison test to determine any significant differences between different groups of hydrogel formulation. This included analyses between cells seeded on a plastic plate vs. 100% collagen vs. 50/50 C/KTN, differences between Ca-free and Ca+ media, and differences between time points.

## 3. Results

### 3.1. Lysosomal Activity Influenced by KTN and Increased Calcium Concentration

**Figure 2:**
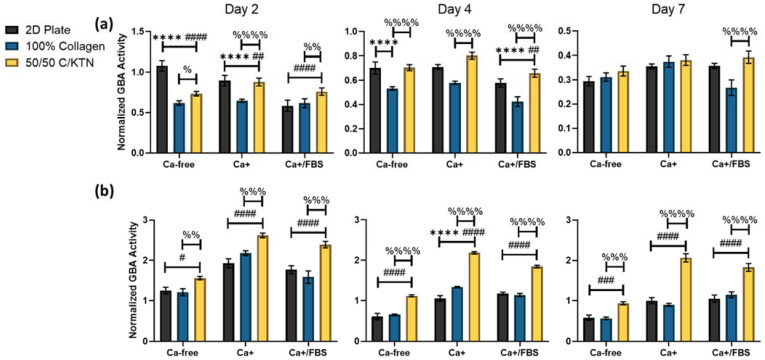
GBA activity of immortalized and primary keratinocytes influenced by KTN and media supplements. GBA activity was normalized to cell viability. Immortalized cells showed significant increase in GBA activity when KTN was added to the hydrogel, but no increase when calcium and FBS was added to the media (**a**). NHEKs showed significant increase in GBA activity when KTN was added to the hydrogel, with increases also observed with addition of calcium and FBS (**b**). GBA activity is displayed as weighted average ± SD. Two-way ANOVA was performed with n = 4 for 3 separate experiments. The different symbols (*, #, %) correspond to the comparisons and the number of symbols correspond to the p-value for each comparison: * = *p* < 0.05, ** = *p* < 0.01, *** = *p* < 0.001, **** = *p* < 0.0001 and * = 2D plate vs. 100% collagen, # = 2D plate vs. 50/50 C/KTN, % = 100% collagen vs. 50/50 C/KTN.

Cell viability and GBA activity were measured, with the latter being normalized to the cell viability at days 2, 4, and 7 post-calcium switch. Ker-CT cells showed no observable increase in GBA activity when calcium was added, as shown in Figure 2a and Table 2. This is possibly due to the immortalized cells behaving similar to cancer cells, which constantly undergo lysosomal biogenesis compared to primary cells [43]. In a previous study on HaCaT cells, an immortalized keratinocyte cell line derived from a tumor showed no increased lysosomal activity when compared to cells activated by calcium [44]. Therefore, their differentiation is not apparent with increase in GBA activity. However, significant (*p* < 0.01) increases in GBA activity were observed when KTN was added to the collagen hydrogel, as observed in Figure 2a, at days 2 and 4 for all conditions of culture media. The 50/50 C/KTN hydrogels had significantly higher GBA activity than 100% collagen hydrogels, indicating that the presence of KTN increased keratinocyte differentiation.

Primary cells (NHEKs) showed a significant (*p* < 0.001) increase in GBA activity when calcium was added to the growth media, with a further rise when FBS was added (Table 2). In addition, there was significant increase for each time point and each medium when KTN was added to the hydrogel when compared to 100% collagen hydrogels (Figure 2b). This can be observed at each time point, even without the addition of calcium to the medium. This increased GBA activity is indicative of lysosomal biogenesis, showing that there is a direct relationship between KTN and increased differentiation.

**Figure 3:**
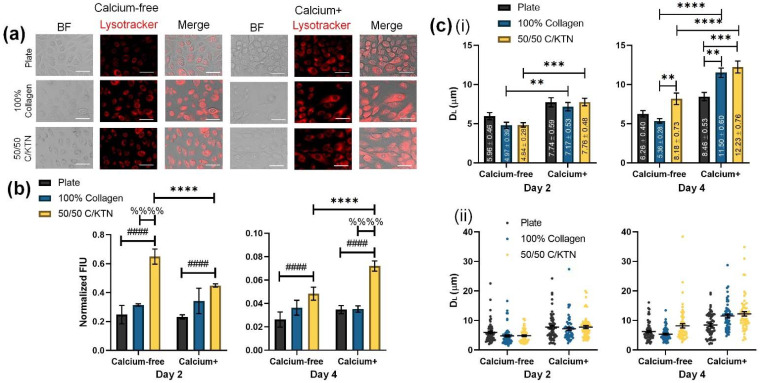
LysoTracker lysosome staining of proliferative and differentiated keratinocytes. Day 2 and day 4 keratinocytes seeded on either a plate or a hydrogel were stained with LysoTracker DND-99 dye to visualize lysosomal distribution, with day 2 shown (**a**). Fluorescence intensity units (FIUs) of stained lysosomes were measured and normalized to cell viability at days 2 and 4 post-calcium switch (**b**), with a significant increase observed for 50/50 C/KTN hydrogels compared to the plate and 100% collagen samples with and without calcium (n = 4, weighted average ± SD; * = day 2 vs. 4 for 50/50 C/KTN, # = 2D plate vs. 50/50 C/KTN, % = 100% collagen vs. 50/50 C/KTN). The D_L_ (**c**) at day 2 showed no significant differences between groups, but increased when calcium was added to the medium (**i**). Significant increases in D_L_ were observed with 50/50 C/KTN when compared to the plate and 100% collagen at day 4 (n = 60, average ± SEM). Each measurement of D_L_ is also plotted to show the distribution (**ii**). Two-way ANOVA was performed for both normalized FIU and D_L_; for all plots. The different symbols (*, #, %) correspond to the comparisons and the number of symbols correspond to the p-value for each comparison: * = *p* < 0.05, ** = *p* < 0.01, *** = *p* < 0.001, **** = *p* < 0.0001; * = 2D plate vs. 100% collagen, # = 2D plate vs. 50/50 C/KTN, % = 100% collagen vs. 50/50 C/KTN. Scale bar = 100 µm.

In addition to the increased GBA activity, the lysosomal distribution and expression also increased when keratinocytes were seeded on top of 50/50 C/KTN hydrogels when compared to the 2D plate and 100% collagen hydrogels. Cells were stained with LysoTracker Red DND-99 dye, which labels the lysosomes of the cells, as shown in Figure 3a. The measured fluorescence intensities of these samples, when normalized to cell viability, indicate that the addition of KTN to the hydrogel significantly increased the lysosomal activity when compared to cells seeded on the plate and 100% collagen for both Ca-free and Ca+ media at days 2 (*p* < 0.001) and 4 (*p* = 0.007 (plate, Ca-free), *p* < 0.0001 (plate, Ca+ and 100% collagen, Ca+)), as shown in Figure 3b. Although D_L_ had no significant differences between groups at day 2 (*p* = 0.4780 to *p* > 0.9999), D_L_ did increase for 100% collagen and 50/50 C/KTN hydrogels when calcium was added to the media (*p* = 0.0044 (100% collagen), *p* = 0.0002 (50/50 C/KTN)) (Table 3). The D_L_ also significantly increased with the addition of KTN when compared to 100% collagen at day 4 (*p* = 0.0007 (100% collagen), and further increased when calcium was included in the media compared to the cells seeded on the plate (*p* < 0.0001) (Figure 3c(i)). Individual measurements are also plotted to show the total distribution of the D_L_, with increased distribution observed with Ca+ samples (Figure 3c(ii)).

### 3.2. Keratinocyte Size Influenced by KTN and Increased Calcium Concentration

**Figure 4:**
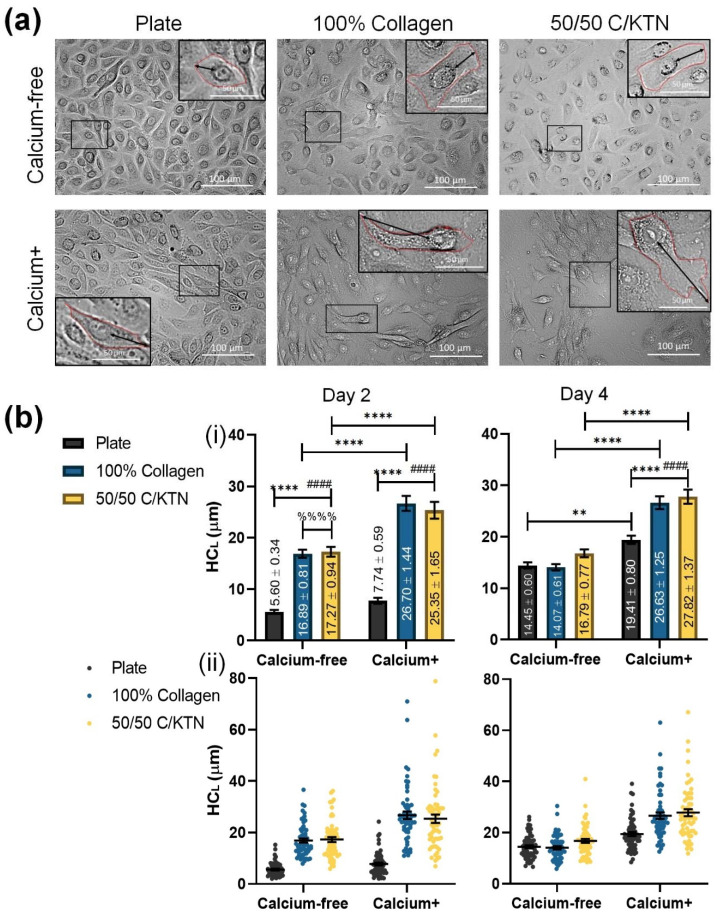
Half-cell length (HC_L_) of keratinocytes under the influence of calcium and hydrogel composition. Day 2 and day 4 keratinocytes seeded on either a plate or a hydrogel were imaged in BF to visualize and measure the half-cell length (**a**). HC_L_ was measured and plotted to compare different groups and media, with significant increases observed for 100% collagen and 50/50 C/KTN when compared to keratinocytes seeded on a plate; displayed as average ± SEM (**b**(**i**)). Each measurement of HC_L_ is also plotted to show the distribution (**ii**). Two-way ANOVA was performed and the different symbols (*, #, %) correspond to the comparisons and the number of symbols correspond to the p-value for each comparison: with * = *p* < 0.05, ** = *p* < 0.01, *** = *p* < 0.001, **** = *p* < 0.0001; * = 2D plate vs. 100% collagen, # = 2D plate vs. 50/50 C/KTN, % = 100% collagen vs. 50/50 C/KTN; n = 60.

Keratinocytes become larger during the differentiation process; therefore, the half-cell length was measured to determine whether the addition of KTN in the hydrogel with the combination of calcium in the medium had any effect in increasing cell size in the form of cell length. As shown in Figure 4A, keratinocytes at day 2 begin to appear larger with calcium in the medium. The half-cell length (HC_L_) measured from the BF images at day 2 and 4 post-calcium switch is plotted and compared between the different groups in Figure 4B(i). Cells seeded on a plastic plate had significantly lower (*p* < 0.0001) half-cell lengths than cells seeded on both types of hydrogels, with a significant increase between Ca-free and Ca+ cells observed at day 2 (*p* < 0.0001). At day 2, the half-cell lengths of cells seeded on top of the plate were less than 19 µm (7.74 ± 0.59); thus, based on the half-cell length, these are considered proliferative cells [37]. Keratinocytes seeded on top of the hydrogels under Ca+ media had average half-cell lengths larger than 19 µm at both time points, indicating that they are in the process of differentiation. A significant increase was observed in cells from Ca-free and Ca+ media seeded on top of 100% collagen and 50/50 C/KTN hydrogels at both day 2 and 4. As shown in Figure 4B(ii), culturing in Ca+ medium led to a wider distribution of half-cell length, indicating that the cells are in the process of differentiation and proliferation.

### 3.3. Involucrin and CK14 Expression

CK14 and involucrin expression were measured by employing flow cytometry and images of stained samples. For FACS analysis, all samples and isotype controls were gated with non-stained sample. In general, efficient blocking was achieved to prevent non-specific binding, as seen by the isotype controls, although some non-specific binding was not preventable (Figure S2a). However, when compared to the samples, there was clearly more positive cells compared to each corresponding isotype control. Within the whole cell population, the proliferative and differentiated cell populations were analyzed separately, in addition to the total cell population, as seen by the forward and side scatter plot (Figure S2b). Percent positive cells are plotted in Figure S2c, with almost all cells expressing CK14, although significantly lower CK14-positive cells were observed with cells treated with the Ca+ medium. On the other hand, cells treated with the Ca+ medium had significantly higher involucrin-positive cells when compared to cells treated with the Ca-free medium for all cell populations. As expected, cells in the differentiated population also had more involucrin-positive cells than the proliferative population. In addition, 50/50 C/KTN hydrogels in the Ca+ medium had significantly higher involucrin-positive keratinocytes than 100% collagen hydrogels for all cell populations, including the total cell population.

**Figure 5:**
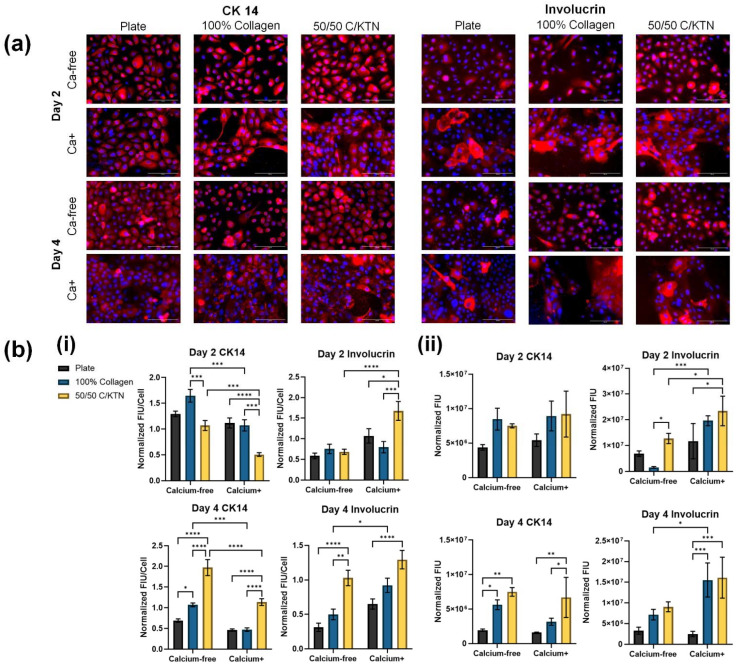
CK14 and involucrin staining and analysis of 2D monolayer samples. Keratinocytes seeded on the plate, 100% collagen hydrogels, and 50/50 C/KTN hydrogels were fixed, permeabilized, and stained for CK14 and involucrin at days 2 and post-calcium switch, with half of the samples still cultured in Ca-free medium (**a**). Images were analyzed (**b**) by CTCF of the marker (**i**) and the total fluorescence intensity per image (**ii**), which was normalized to the DAPI CTCF and the DAPI cell count, respectively. In general, CK14 was expressed significantly more in Ca-free samples when cells were analyzed individually. Involucrin expression was increased with Ca+ media with significantly higher involucrin expression observed with cells seeded on top of 50/50 C/KTN hydrogels. FIU/cell and total FIU is displayed as average ± SEM and SD, respectively. Two-way ANOVA was conducted with n = 3; * = *p* < 0.05, ** = *p* < 0.01, *** = *p* < 0.001, **** = *p* < 0.0001. Scale bar = 100 µm.

Images analyzed for fluorescence intensity of CK14 and involucrin showed similar results as the FACS analysis. Fluorescence images, as shown in Figure 5a, were processed using ImageJ software, and the fluorescence intensity units (FIUs) per cell and per image are plotted at days 2 and 4 post-calcium switch. From what can be observed from the images, the visually larger cells cultured in the Ca+ medium show higher fluorescence intensity of involucrin for both Ca-free and Ca+ media, with larger cells present in the Ca+ medium, as discussed earlier. Similar results were observed for both types of analyses (CTCF and total fluorescence/image), with significantly higher involucrin intensities observed for keratinocytes cultured in the Ca+ medium, and higher involucrin intensity with keratinocytes seeded on top of 50/50 C/KTN hydrogels. CK14 intensity significantly decreased with keratinocytes cultured with the Ca+ medium (Figure 5b), with significantly lower CK14 intensities observed at day 4 compared to day 2 when cultured in the Ca+ medium (Table 4).

## 4. Discussion

Most organotypic skin models use collagen as the extracellular matrix (ECM) for the dermal layer, in which fibroblasts are seeded, and upon which keratinocytes are seeded to form the epidermis with exposure to the ALI. Keratin is the primary protein present in the epidermis and is produced by keratinocytes, comprising approximately 90% of the mass of the epidermis [17]. Although keratin is not present in the dermal layer, we wanted to determine whether keratin promotes the differentiation of keratinocytes when it is included in the ECM upon which the keratinocytes are seeded. Because keratin is naturally found in the epidermis, we employed a 50/50 w%/w% collagen/keratin hydrogel, which allowed the spreading and proliferation of NHDFs, as previously shown by the Rylander laboratory [36].

We hypothesized that the presence of keratin, without fibroblasts, and the use of the ALI would promote differentiation of keratinocytes with greater lysosome (GBA) activity and expression of differentiation markers, including involucrin. As demonstrated, GBA activity for immortalized (Ker-CT) and primary keratinocytes significantly increased when KTN was added to the hydrogel. In a previous study conducted by Mahanty et al., a twofold increase in GBA activity was observed with the addition of calcium in the media with a 2D model of keratinocytes seeded in a well-plate [37]. We documented similar results with the primary cells seeded on hydrogels, particularly for those seeded on 50/50 C/KTN hydrogels. We observed significant twofold increases in GBA activity with 50/50 C/KTN hydrogels compared to the 2D plate and 100% collagen hydrogels. However, immortalized keratinocytes showed no significant increases in GBA activity when calcium was added, possibly because immortalized cells behave similar to cancer cells, which constantly undergo lysosomal biogenesis as they proliferate [43,47]. Cancer and immortalized cells differ in normal metabolism to maintain uncontrolled growth and senescence, resulting in rapid depletion of cellular nutrients and damaged organelles, therefore increasing lysosome activity [47]. The rise in calcium concentration did not have any major effect in increasing lysosomal biogenesis for immortalized keratinocytes. However, the addition of KTN to the hydrogel did significantly increase the GBA activity of both primary and immortalized keratinocytes when compared to 100% collagen hydrogels, indicating that KTN increases keratinocyte differentiation. Previous studies on immortalized HaCaT keratinocytes, which are derived from a tumor, showed no increased in lysosome activity with greater calcium [44].

To confirm that the KTN was increasing the differentiation of the immortalized keratinocytes, half-cell length and dispersion length (D_L_) and total fluorescence intensity of lysosomes was measured at days 2 and 4 post-calcium switch with controls for culture in Ca-free media. With increased keratinocyte differentiation, the D_L_ of keratinocytes cultured on top of 100% collagen and 50/50 C/KTN hydrogels increased significantly at day 4 when compared to cells cultured on 2D plates, with overall increase in D_L_ in Ca media. Similar results were observed by Mahanty et al., in which keratinocytes cultured in media containing calcium had increased cell size compared to keratinocytes cultured in Ca-free media [37]. This terminal differentiation of keratinocytes into corneocytes are correlated with increasing size, as keratinocytes in vivo enlarge as they progressively move up through the basal layer into the granular layer of the epidermis [28]. This may be due to the enlargement of the cytoplasm as the cell produces more proteins such as involucrin and filaggrin to prepare for terminal differentiation [48,49]. Keratinocyte differentiation can also lead to the dispersion of lysosomes as increased lysosomal biogenesis occurs in response to the increased intracellular calcium levels. To avoid programmed apoptosis, membrane-bound organelles are likely to accommodate for the excess intracellular calcium, leading to lysosomal dispersion near the cell surface instead of near the nucleus [37,45,46,50]. In addition, culture on hydrogels, a 3D surface, increased the FIU of lysosomal activity, with further significant increase in FIU with 50/50 C/KTN samples compared to 100% collagen hydrogels. This indicated an increased quantity of lysosomes in the cell. The importance of a 3D ECM structure for keratinocyte differentiation was also confirmed with cell size correlating with increased calcium concentration and culture on both 100% collagen and 50/50 C/KTN hydrogels. Keratinocytes cultured on a 2D well surface were small enough to still be considered proliferative at day 2 (<19 µm) [37,48] when cultured in both Ca-free and Ca+ media, whereas keratinocytes cultured on hydrogels in both types of media were already considered differentiated with average cell size threefold greater than those seeded on the plate. As average keratinocyte cell size increased over time and with culture on hydrogels and Ca+ media combined, the dispersion of cell size increased, indicating the presence of both proliferating and differentiating keratinocytes at different levels.

Involucrin, a terminal differentiation marker, is expressed in the cornified envelope of human skin and only expressed in enlarging keratinocytes that are in the process of terminal differentiation [49,51,52]. We were able to demonstrate in our hydrogel monolayer model that the inclusion of KTN without the influence of the ALI and the fibroblast feeder layer led to the increase in involucrin-positive cells and involucrin expression as determined by FACS and fluorescence intensity analysis, respectively. We decided to analyze two different cell populations in our forward and side scatter plot, following methods described by Sanz-Gomez et al. [53]. These two populations of proliferating and differentiating cells can be identified as proliferative basal keratinocytes, and have lower and homogenous FSC and SCC points without much dispersion, whereas the differentiating population can be identified as a heterogenous population [53]. We were able to identify these populations with keratinocytes seeded on top of 100% collagen and 50/50 C/KTN hydrogels. As expected, almost all cells in all conditions were positive for CK14 expression, a marker for keratinocyte proliferation, with a small percentage of cells staining positive for involucrin with FACS analysis. However, when analyzing the expression of CK14 by measuring fluorescence intensity, CK14 expression was significantly lower in samples when cultured in Ca+ conditions, indicating that the calcium was inducing more differentiation and less proliferation of keratinocytes compared to those cultured in Ca-free conditions. Keratinocytes cultured on 50/50 C/KTN hydrogels had significantly higher percentages of involucrin-positive cells in Ca+ culture conditions compared to 100% collagen and Ca-free culture conditions. In addition, the normalized fluorescence intensity also increased with Ca+ culture conditions, with visibly larger cells exhibiting greater fluorescence than smaller cells, validating that the larger cells are differentiating.

There are several limitations to the current study. Complete differentiation of keratinocytes into corneocytes in native human skin take on average 14 days, during which the dead cell layer of your skin turns over [17,20,54,55]. Therefore, most studies employ 14–21-day cultures of keratinocyte on the 3D surface at the ALI. Our study only investigated keratinocyte differentiation submerged in culture medium for a maximum of 7 days. Although we were unable to culture at the ALI for longer than 7 days, we were able to demonstrate that KTN was able to increase differentiation of Ker-CT cells without the influence of the ALI and longer culture. In addition, we only compared our results to primary keratinocytes for the GBA activity, in which we were unable to show increased GBA activity with Ker-CT cells with the addition of calcium to the media. We believe this is due to the Ker-CT cells possibly undergoing lysosomal biogenesis due to constant proliferation [43,44]. However, a recent study by Tito et al. demonstrated increased GBA activity with HaCaT cells when treated with *Triticum vulgare,* a plant-derived treatment known to accelerate wound healing [56]. This suggests that an appropriate inducer for increasing GBA activity due to differentiation may be needed to overcome the GBA activity reading from constant proliferation.

In relation to this study, KTN may be helpful in improving wound healing by increasing the differentiation of keratinocytes, therefore leading to increased skin renewal. Previous studies have reported the clinical uses of keratin as a therapeutic dressing to increase wound healing in chronic wounds and burn injuries [57,58,59,60,61]. In future studies, we aim to test KTN in the presence of an organotypic model to determine whether the addition of KTN further enhances differentiation. Further investigation into the clinical aspect of KTN in improving wound healing in relation to increasing differentiation will also be needed to understand the full potential of keratin as a therapeutic.

## 5. Conclusions

In this study, we demonstrate the presence of keratin in the ECM promoted keratinocyte differentiation without the use of a fibroblast feeder layer and the ALI, traditionally used on 3D organotypic skin models. The presence of KTN increased lysosomal activity, with increases in GBA activity and dispersion length of lysosomes throughout the cytoplasm in immortalized keratinocytes, indicating keratinocyte differentiation. More importantly, the inclusion of KTN concentration increased the expression of involucrin, a terminal differentiation marker that is normally seen in native skin, with further increased differentiation observed with calcium included in the culture media. Although it is still important to include the ALI and the fibroblast feeder layer to represent human skin, the addition of KTN in future skin models in combination with these other inducers of keratinocyte differentiation may create a more representative skin model. Furthermore, KTN may be beneficial in improving wound healing by increased keratinocyte turnover through keratinocyte differentiation.

## Figures and Tables

**Table 1:** KTN concentration and total protein concentration for each type of hydrogel. The concentration of collagen remains constant.

Hydrogel	Collagen Concentration	KTN Concentration	wt%/vol%
100% Collagen	4.0 mg/mL	0.0 mg/ml	4.0
50/50 C/KTN	4.0 mg/mL	4.0 mg/ml	8.0

**Table 2:** Comparison of GBA activity between different media within each sample group. GBA activity was normalized to cell viability. For most samples, especially for primary NHEKs, addition of in ^c^calcium increased the GBA activity; addition of FBS also further increased the GBA activity. Two-way ANOVA was performed with n = 4 with 3 separate experiments. * = *p* < 0.05, ** = *p* < 0.01, *** = *p* < 0.001, **** = *p* < 0.0001, ns = not significant.

Sample Group	Medium	*p*-Value
Plate, Ker-CT	Ca-free vs. Ca+	Day 2 (*p* < 0.0001)—**** Day 4 (*p* > 0.9999)—ns Day 7 (*p* = 0.0392)—*
	Ca-free vs. Ca+/FBS	Day 2 (*p* = 0.0216)—* Day 4 (*p* < 0.0001)—**** Day 7 (*p* = 0.0317)—*
	Ca+ vs. Ca+/FBS	Day 2 (*p* < 0.0001)—**** Day 4 (*p* < 0.0001)—**** Day 7 (*p* > 0.9999)—ns
100% Collagen, Ker-CT	Ca-free vs. Ca+	Day 2 (*p* = 0.9925)—ns Day 4 (*p* = 0.2850)—ns Day 7 (*p* = 0.0329)—*
	Ca-free vs. Ca+/FBS	Day 2 (*p* > 0.9999)—ns Day 4 (*p* < 0.0001)—*** Day 7 (*p* = 0.2709)—ns
	Ca+ vs. Ca+/FBS	Day 2 (*p* = 0.9859)—ns Day 4 (*p* < 0.0001)—**** Day 7 (*p* = 0.0002)—***
50/50 C/KTN, Ker-CT	Ca-free vs. Ca+	Day 2 (*p* = 0.0017)—* Day 4 (*p* = 0.0005)—*** Day 7 (*p* = 0.2412)—ns
	Ca-free vs. Ca+/FBS	Day 2 (*p* = 0.9961)—ns Day 4 (*p* = 0.3243)—ns Day 7 (*p* = 0.0724)—ns
	Ca+ vs. Ca+/FBS	Day 2 (*p* = 0.0161)—* Day 4 (*p* < 0.0001)—**** Day 7 (*p* = 0.9984)—ns
Plate, NHEK	Ca-free vs. Ca+	Day 2 (*p* < 0.0001)—**** Day 4 (*p* < 0.0001)—**** Day 7 (*p* < 0.0001)—****
	Ca-free vs. Ca+/FBS	Day 2 (*p* < 0.0001)—**** Day 4 (*p* < 0.0001)—**** Day 7 (*p* < 0.0001)—****
	Ca+ vs. Ca+/FBS	Day 2 (*p* = 0.4885)—ns Day 4 (*p* = 0.0526)—ns Day 7 (*p* = 0.9928)—ns
100% Collagen, NHEK	Ca-free vs. Ca+	Day 2 (*p* < 0.0001)—**** Day 4 (*p* < 0.0001)—**** Day 7 (*p* = 0.0007)—***
	Ca-free vs. Ca+/FBS	Day 2 (*p* = 0.0020)—** Day 4 (*p* < 0.0001)—**** Day 7 (*p* < 0.0001)—****
	Ca+ vs. Ca+/FBS	Day 2 (*p* < 0.0001)—**** Day 4 (*p* = 0.0006)—*** Day 7 (*p* = 0.0138)—*
50/50 C/KTN, NHEK	Ca-free vs. Ca+	Day 2 (*p* < 0.0001)—**** Day 4 (*p* < 0.0001)—**** Day 7 (*p* < 0.0001)—****
	Ca-free vs. Ca+^/^FBS	Day 2 (*p* < 0.0001)—**** Day 4 (*p* < 0.0001)—**** Day 7 (*p* < 0.0001)—****
	Ca+ vs. Ca+/FBS	Day 2 (*p* = 0.1154)—ns Day 4 (*p* < 0.0001)—**** Day 7 (*p* = 0.0173)—*

**Table 3:** D_L_ of measured lysosomes tagged with LysoTracker staining. Measurements are displayed as average ± SEM with n = 60.

Sample Group	Medium	D_L_ (µm)
Plate	Ca-free	Day 2—5.96 ± 0.45 Day 4—6.26 ± 0.40
	Ca+	Day 2—7.74 ± 0.59 Day 4—8.46 ± 0.53
100% Collagen	Ca-free	Day 2—4.97 ± 0.39 Day 4—5.36 ± 0.28
	Ca+	Day 2—7.17 ± 0.53 Day 4—11.5 ± 0.60
50/50 C/KTN	Ca-free	Day 2—4.84 ± 0.28 Day 4—8.18 ± 0.73
	Ca+	Day 2—7.76 ± 0.48 Day 4—12.23 ± 0.76

**Table 4:** Comparison of fluorescence intensity of differentiation markers between time points within each group. Two-way ANOVA was performed with n = 4 with 3 separate experiments. * = *p* < 0.05, ** = *p* < 0.01, *** = *p* < 0.001, **** = *p* < 0.0001, ns = not significant.

Sample Group	Media	Day 2 vs. Day 4
Plate	Ca-free—CTCF	CK14 (*p* = 0.0013)—** Involucrin (*p* = 0.2088)—ns
	Ca+—CTCF	CK14 (*p* < 0.0001)—**** Involucrin (*p* = 0.4185)—ns
	Ca-free—total fluorescence	CK14 (*p* = 0.0235)—* Involucrin (*p* = 0.0349)—*
	Ca+—total fluorescence	CK14 (*p* = 0.0235)—ns Involucrin (*p* = 0.2073)—ns
100% Collagen	Ca-free—CTCF	CK14 (*p* = 0.0025)—** Involucrin (*p* = 0.2609)—ns
	Ca+—CTCF	CK14 (*p* < 0.0001)—**** Involucrin (*p* = 0.9924)—ns
	Ca-free—total fluorescence	CK14 (*p* = 0.0079)—** Involucrin (*p* = 0.0012)—**
	Ca+—total fluorescence	CK14 (*p* = 0.0433)—* Involucrin (*p* = 0.8726)—ns
50/50 C/KTN	Ca-free—CTCF	CK14 (*p* < 0.0001)—**** Involucrin (*p* = 0.0559)—ns
	Ca+—CTCF	CK14 (*p* < 0.0001)—**** Involucrin (*p* = 0.4680)—ns
	Ca-free—total fluorescence	CK14 (*p* > 0.9999)—ns Involucrin (*p* = 0.0272)—*
	Ca+—total fluorescence	CK14 (*p* = 0.6690)—ns Involucrin (*p* = 0.4151)—ns

## Data Availability

The data presented in this study are available in the article and the Supplementary Material.

## Supplementary Materials

The following supporting information can be downloaded at: https://www.mdpi.com/article/10.3390/bioengineering9100559/s1. Figure S1: Cell viability of keratinocytes, Figure S2: Flow cytometry analysis of CK14 and involucrin expression at day 4 post-calcium switch.
