# Supplementary material for: Keratin Promotes Differentiation of Keratinocytes Seeded on Collagen/Keratin Hydrogels
Source: Bioengineering (Basel). 2022 Oct 15;9(10):559. doi: 10.3390/bioengineering9100559 (PMC9598618; doi:10.3390/bioengineering9100559)
Supplement: Supplementary file 1 [file bioengineering-09-00559-s001.zip › bioengineering-1926687-supplementary.pdf]

Supplementary Data

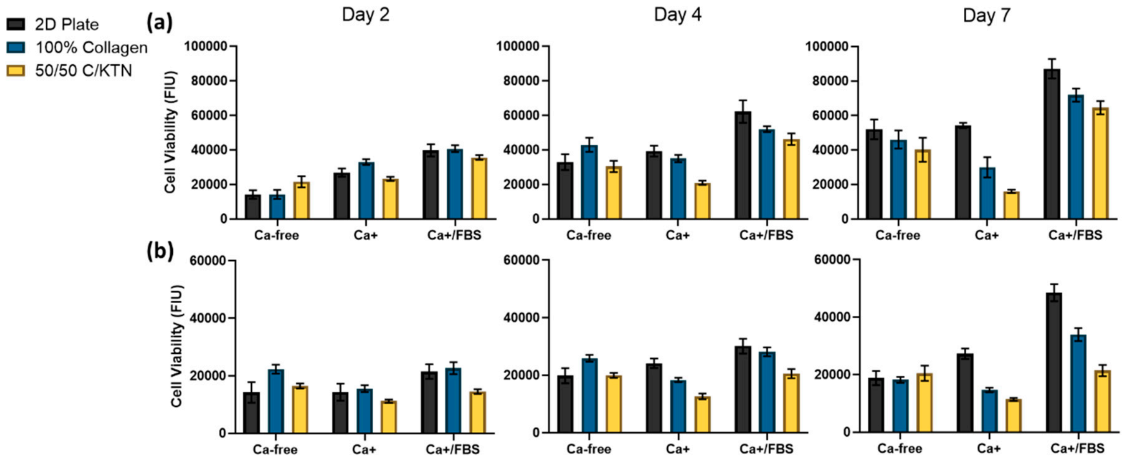

Figure S1: Cell viability of keratinocytes. Cell viability was measured for Ker-CT cells (**a**) and NHEKs (**b**) using the Cell Titer Blue viability assay. This data was used to normalize the GBA assay results. An increasing trend in cell viability at longer culture time can be observed at day 7.

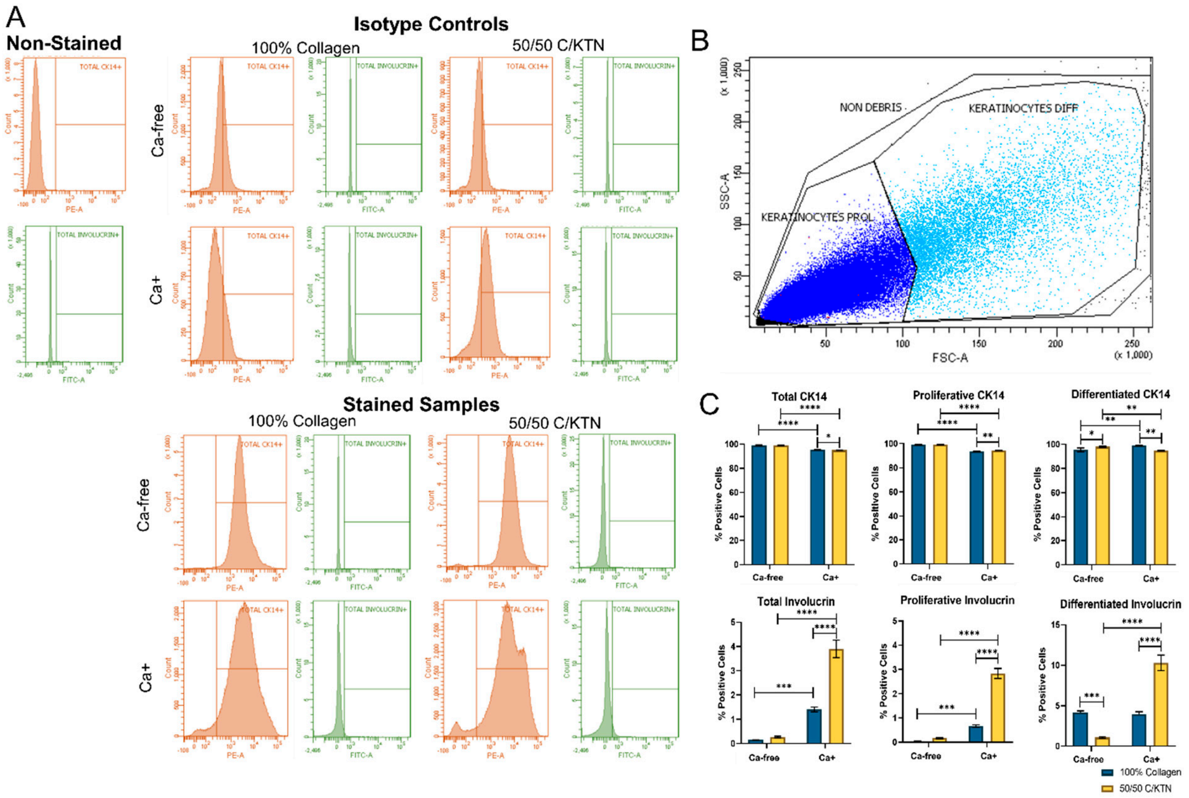

Figure S2: Flow cytometry analysis of CK14 and involucrin expression at day 4 post-calcium switch. Histogram plots of each sample, including non-stained and isotype controls (**A**), with all samples and isotype controls being gated to the non-stained sample. Two populations of keratinocytes within the forward and side-scatter plot were analyzed, one being the smaller proliferative cells and the other being the larger differentiated cells (**B**). The total population was also analyzed as a whole. Percent positive cells for CK14 and involucrin were plotted for each group with significantly higher involucrin expression observed with keratinocytes seeded on 50/50 C/KTN hydrogels with calcium added to the media (**C**). Almost all cells were positive for CK14. Two-way ANOVA was conducted with n=3; * = $p < 0.05$, ** = $p < 0.01$, *** = $p < 0.001$, **** = $p < 0.0001$.
